# Scorpions: A Presentation

**DOI:** 10.3390/toxins6072137

**Published:** 2014-07-21

**Authors:** Max Goyffon, Jean-Nicolas Tournier

**Affiliations:** 1 Department RDDM, National Museum of Natural History, 57 rue Cuvier, 75005 Paris, France; 2 Unit “Interactions Host-Pathogen”, IRBA, BP 73, 91223 Brétigny-sur-Orge, France; E-Mail: jntournier@gmail.com or jean-nicolas.tournier@pasteur.fr; 3 Laboratory “Pathogeny of Bacterial Toxi-Infections”, Pasteur Institute, 28 rue du Dr-Roux, 75725 Paris Cedex 15, France; 4 Ecole du Val-de-Grâce, 1 place Alphonse Laveran, 75005 Paris, France

**Keywords:** scorpions, biology, venom toxins, defensins

## Abstract

Scorpions, at least the species of the family Buthidæ whose venoms are better known, appear as animals that have evolved very little over time. The composition of their venoms is relatively simple as most toxins have a common structural motif that is found in other venoms from primitive species. Moreover, all the scorpion venom toxins principally act on membrane ionic channels of excitable cells. The results of recent works lead to the conclusion that in scorpions there is a close relationship between venomous function and innate immune function both remarkably efficient.

## 1. Scorpions: Biology and Physiology

Scorpions belong to the phylum Arthropods, subphylum Chelicerata. Appearing at the Silurian (450 millions years), these arthropods are considered as the most ancient terrestrial animals [1]. They represent an order comprising only nearly 2000 species (compared to 44,000 spider species or about 1,000,000 insects). All the species of this small and tiny order are venomous. Moreover they possess some unique characteristics regarding their physiology as well as pertaining to the composition and the effects of their venoms. These characteristics principally concern the family Buthidæ, the medically most important and remarkable. The non-Buthids (previously “Chactoids”) are medically less important and, hence, studied to a lesser extent. All scorpions have a circulating hemocyanin, a respiratory cuproprotein, which is able to reversibly bind with oxygen. The scorpion hemocyanins possess a triple enzymatic function, pseudo-catalasic, peroxydasic and superoxide-dismutasic [2,3,4]. The scorpion hemocyanins are also likely to have an antimicrobial activity due to these multiple oxidative enzymatic functions [5]. 

The family Buthidæ presents some surprising capacities of resistance to environmental stressors [6]: resistance to dehydration, to microbial infections, and to ionizing radiations (gamma rays). Its resistance to dehydration is important as an important increase in blood osmolarity of at least 600 mosmoles and more, can take place, which is twice or more the usual osmolarity. The good resistance to bacterial infections is probably due to the constitutive presence of defensins in the blood [7]. The resistance to ionizing radiations is not yet explained [8]. 

Scorpions are usually gendered, *i.e.*, males or females. Mating usually occurs in the spring season, when temperatures rise and days get longer, in the Northern hemisphere in the month of April. Most often the gestation lasts three or four months depending on the temperature and on the food resources. For African species of the genus *Androctonus*, females must undergo a thermal peak of 34 °C before parturition. Some species are parthenogenetic: males can be found but they are very rare (species of the genera *Buthacus* and *Tityus* for example). Pullus that hatch have some morphological differences to the adults. Just after birth, they climb up the back of their mother and molt simultaneously after a few days. They get rid of some sort of common envelope. After this they have the morphology of miniature adults differing only by the absence of sexual maturity. After a series of molts, usually seven, they reach adulthood and are then able to reproduce. Some rare post-imaginal molts are observed. 

Some scorpion species can easily adapt to anthropic environments provided they can find food, generally other small arthropods (insects, millipedes, and woodlice). If the scorpion species is harmless, annoyance is modest, but if it is dangerous, medical risk is present, especially for children. In North Africa, scorpions often live in gardens and can enter homes: the gardens offer shelter in the form of, for example, certain plants, such as prickly pears or climber plants. Additionally, these houses are sometimes not well maintained, with crevices in their walls in which scorpions can hide. Sometimes, urban hygiene is neglected, which can indirectly provide protection and food for scorpions, which can lead to “domestic stings” as a consequence. However, it is possible that an urban environment favors the development of new scorpion populations. In France, the construction of the Midi Channel in the 18th century between the Mediterranean Sea and the Atlantic Ocean, along the river Garonne, favored the migration of the harmless species *Euscorpius flavicaudis* from the Mediterranean coast to the Atlantic coast, and allowed it to spread in the cities along the river Garonne, from Sete to Bordeaux, due to the barges which involuntarily transported scorpions. Bordeaux is now colonized with this little black scorpion sometimes found in wine cellars. In Argentina and in Brazil, the situation is quite different. Large cities (Buenos Aires, Argentina, Brasilia or Sao Paulo, Brazil) are inhabited by parthenogenetic species of the dangerous genus *Tityus* sp., *T. trivittatus* in Argentina, and *T. serrulatus* in Brazil. These species are consumers of cockroaches settled in buildings, and so food is abundant. Scorpions were probably involuntarily transported by various vehicles (cars, trucks). And now, as all the *Tityus* species are considered dangerous, Brasilia as well as Buenos Aires are faced with a real problem of public health. 

Scorpions have small ventral appendages, combs, near the genital orifice, which is the remarkable characteristic of the order. For some authors the presence of combs is a sufficient character to consider this order to be monophyletic. The combs, articulated and mobile, are provided with tactil receptors and chemoreceptors [9]. However, they do not seem to have receptors sensitive to humidity as presumed sometimes. Track combs and pulmonary stomata are visible for all the Carboniferous species, which are therefore considered to have pulmonary breathing. In the Silurian, neither pulmonary stomata nor the tracks of combs are visible. Perhaps these ancient scorpions were once marine organisms. Hence, there has often been a confusion of Gigantostracea and scorpions because of their external morphological similarity. The Gigantostracea were large marine animals (1.50 m and more). However, the absence of visible combs on fossils currently available does not allow them to be considered as scorpions. 

All the scorpions fluoresce under UV light. The whole body is fluorescent, not just certain parts of the body (joints) as is the case for, e.g., “camel spiders” (Solifugæ). The nature of the fluorescent pigments is known: two pigments have been identified, a derivative of carboline and a derivative of coumarine [10]. Exuviæ are fluorescent as well, and pullus do not produce fluorescence prior to their common molt. This feature allows easy the collection of scorpions, as they are nocturnal animals. The UV lamp is also a measure of security in areas harbouring dangerous species. The physiological role of this fluorescence is still discussed. 

Internal insemination is performed after a delay, which is sometimes short (about 15–20 min) or often long (more than 24 h), a time during which the male, taking the female by the claws with its claws, drives the female into a sort of dance. The male puts down a spermatophore containing a small pocket with the sperm on a plane surface, then it puts the female onto the spermatophore which penetrates the genital tract of the female. When breeding, a female who has been mated is no longer attractive to males (*Androctonus* sp.). In nature, it is not clear whether this phenomenon exists, given the density of scorpions observed in some territories, which implies the existence of several litters for a female species during its lifetime. So, it is known that female scorpions can keep some living sperm reserves in their genital tract. The number of pullus of a litter varies from one species to another, from sometimes about twenty (*Euscorpius* sp., Euscorpiidæ), to sometimes slightly more (*Pandinus* sp., Scorpionidæ) to up to more than one hundred pullus (*Androctonus* sp., Buthidæ). All the pullus appear in the same night (*Androctonus* sp.), sometimes during several successive nights (*Pandinus* sp.). Adult sizes vary according to the species. Some species are small, about 1 cm (*Microbuthus*, *Orthochirus*, Buthidæ), other can reach 10 cm or more (*Androctonus*). The giant of the scorpions, *Pandinus imperator*, is also the giant of terrestrial arthropods with a maximal length of 25 cm. The lifetime of scorpions is often inversely proportional to their size. The duration of intervals between moltings is also variable, according to temperature and nutrition. Species of the smaller genera, such as *Euscorpius* sp., have a life span of one or two years, on the other hand, *Androctonus* sp., which have a relatively large size, live for about 8 years, and *Pandinus imperator* are likely to live for more than 10 years. 

The determination of scorpions is not easy. Morphological characters can indeed give rise to small but continuous changes, especially when the distribution of the species is large, which is not frequent because the rate of endemicity of scorpion species is high. The case of the European and North-African species *Buthus occitanus* is a good illustration. In the countries of North Africa, some morphological differences between the western forms (Morocco) and the eastern forms (Tunisia) suggest that they might be different species. However when collecting specimens in neighboring stations, a continuous gradient of variations is observed and the definition of species or subspecies is very difficult or even impossible. This is why it is important that the systematics of scorpions relies on molecular data that have contributed to the identification of many new families. Thus, according to the authors, the previous number of six or seven families should be increased to fifteen or more [9,11,12]. However, the authors’ opinion is that the Buthidæ family remains the most important both in number of species and best known species from a medical point of view [13]. Because of the large number of species, some attempts for dividing this large family into subfamilies have been proposed. The first attempt as a description of subfamilies based on biogeographical criteria, this, however, was not further persued. A clearer attempt based on both morphological and immunochemical criteria was unsuccessful, probably due to the too small sample size of scorpions [14].

It is rare to observe epizootic diseases in scorpion breedings as it can be seen, for example, in “breeding forages” of crickets. Scorpions of the Buthidæ family are remarkably resistant to bacterial infections. This resistance was discovered by Morel [15] and then confirmed by Cociancich *et al.* [7]. It was interpreted as a result of the constitutive presence of defensins in scorpion blood. However, with the resistance of scorpions being far higher than the resistance of insects, it is likely that other unknown antibacterial factors are involved. However, scorpions are sensitive to certain rickettsiæ, which are easily transmitted from an infected mother to her offspring. Breedings can also be invaded by small mites whose presence could be a sign of bad physiological state.

## 2. Scorpions: The Venomous Function

### 2.1. The Venom Gland

The venom gland is derived from a differentiation of the last segment of the post-abdomen (telson), in a post-anal position. It does not develop from a pre-existing organ into a secondarily differentiated venom gland, as is the case in animals with an oral venomous apparatus (spiders, snakes) or with a glandular skin apparatus (fish, platypus). The telson is internally covered by a simple endoderm without specific differentiation and harbours two terminal nerve branches of the ventral nervous chain. Given these facts, it can be expected that the composition of the venom is simple in so ancient animals, with a similar stucture of the different toxins, which can also be found in the venom of other ancient venomous animals. Indeed, the structure and the effects of the toxins of Buthidæ scorpions are similar.

### 2.2. The Structure of the Venom Toxins

All scorpion toxins are composed of a short chain of aminoacid residues. There are short toxins (30–40 aminoacids) and long toxins (about 60 aminoacids). Short toxins possess a common stuctural motif termed “CSab”, “Cystein Stabilized alpha-beta” motif, consisting of three disulfide bridges, two of which connect the alpha helix and the beta sheet, the third connecting the alpha helix and the C-terminus of the proteic chain. Long toxins contain a fourth disulfide bridge, which connects the two extremities of the proteic chain. Hence, the core of long toxins is identical to the short toxin as it also contains the CSab motif. To date, long toxins seem to have been found only in Buthidæ venoms.

The pattern CSab is not only found in scorpion venom toxins but also in a particular type of antimicrobial peptide (AMP), the defensins, always present in the blood of scorpions, which is considered to be “constitutive”. Generally, they are inducible in insects, but insect defensins and scorpion defensins belong to the same molecular group raising the question of a common ancestor in scorpions for the neurotoxins and the defensins [7].

The hemolymph of the North African dangerous scorpion *Androctonus australis* contains an AMP termed “androctonin” with a peptidic chain of 25 aminoacids and two disulfide bridges. The structure of androctonin has a relationship with a curarizing cone venom toxin, the a-neurotoxin SII, which is a blocker of the nicotinic acetylcholinereceptor. It has been observed that the androctonin and the cone venom toxin SII both have a comparable affinity to the *Torpedo* nicotinic acetylcholine receptor [16]. It must be noted that the short neurotoxins with two disulfide bridges are common in many venoms: apamin and MCD peptide (bees), conotoxines (cones), and sarafotoxins (snakes genus *Atractaspis*). This observation gave rise to the following remark by Zhu *et al.*: “Our finding might also be important in considering toxicity of antibacterial defensins as drugs” [17].

### 2.3. The Venom Effects

The toxicity of the Buthid venoms is due to toxins acting essentially on ionic channels of the membrane cells of the excitable tissues, in particular on nerves for long toxins and on many tissues for short toxins. 

(i) **The long toxins** act on sodium channels and are principally responsible for the envenoming symptomatology. There are two types of long toxins, alpha and beta, according to their mechanism of action. The alpha-type toxins are found almost exclusively in the venom of Paleotropical scorpion species. They inhibit the phase of inactivation of the action potential and bind to Site 3 of the Na^+^ channel. The beta-type toxins are exclusively found in the venoms of Neotropical species. They reduce the excitability threshold of membrane excitable cells and bind to a different site, Site 4 (Table 1). In the Buthidæ venoms, only one type of long toxins is generally found, either alpha or beta. Therefore the scorpion venoms allow the identification of two different (of the seven now recognized) sites on the channel sodium. Mollusc and amphibian venoms can identify other specific sites (Table 1). Generally, insect-toxins are paralyzing. The “flaccid” ones are similar to the alpha-type toxins and provoke a flaccid paralysis, the “spastic” toxins act like mammalian beta-toxins and provoke a spastic paralysis. 

(ii) **The short toxins** are principally active on membrane cell potassium channels or chloride channels and are present in small quantities in the venoms (about 0.2% of the dry venom for the short toxins blocking potassium channels). They are found in all scorpion venoms studied, including both the Buthidæ family and the non-Buthidæ families, and have been extensively studied, as potassium channels are ubiquitously distributed throughout the simplest to the most advanced living beings. Short toxins acting on potassium channels are numerous: a total of over 200 KTx oligonucleotides or peptide sequences isolated from scorpion venoms are referenced in the data bank UniProtKB [18]. They constitute a superfamily of toxins divided into two main families based on their primary structure and their specificity: the voltage-dependant toxins and the ligand-dependant toxins [19]. Several subfamilies are currently distinguished: the a-KT toxins include short and ultra-short toxins (26–40 aminoacids), the b-KTx include long chains (50 aminoacids or more). There are also the particular k-toxins (hefantoxins) from the venom of the Indian Scorpionidæ *Heterometrus fulvipes* [20]. All the toxins blocking potassium channels are cross-linked by three disulfide bridges (rarely four) with the motif CSab, which is also found in defensins. These toxins are devoid of toxicity in mammals unless they are injected by intracerebral route, in which case they provoke convulsions. Many of them can be synthetized chemically or by genetic engineering. The structural flexibility of the toxin blockers of potassium channels and the K^+^ channel are important factors in the specificity of the intractions of the toxin with the channel [21]. In fact, for some authors, it is always a lysine side chain which blocks the K^+^ channels, irrespective of its position in the toxin sequence [22].

**Table 1:** Sites of ligand binding and effect on sodium channels of some toxins and venom toxins according to Legros [13].

Site	Toxins	Effects
1	Tetrodotoxin Saxitoxin μ-Conotoxin GIIIa	Blocking ionic conductance
2	Batrachotoxin Veratridin Aconitin	Permanent activation
3	Scorpion toxins-type	Inhibition of inactivation
4	Scorpion toxins-type	Decrease of activation threshold
5	Brevetoxins Ciguatoxins	Facilitation of activation and Inhibition of inactivation
6	d-Conotoxins	Slowing of inactivation
7	Pyrethroids	Decrease of activation threshold Slowing of inactivation

Among the short toxins, chlorotoxin is very interesting because of its possible medical applications. It was initially discovered by DeBin & Strichartz [23,24] in the venom of the African and Oriental dangerous scorpion *Leiurus quinquestriatus*, but was also identified in the venom of other Buthidæ [25]. Rapidly, the therapeutic interest of the scorpion venom chlorotoxin was reported [26], and the chlorotoxin was synthetized under the name of TM-601 [27]. Glioma are dangerous cerebral tumors with a median survival time of around 15 months with usual recurrences after a surgical resection followed by a radiotherapy [28]. A patent requested in 1996 was accepted in 2013 [29], and Phase 1 clinical tries were undertaken several years ago [30]. The TM-601 also possesses an antiangiogenic effect, which is important as it also reduces the secretion of metalloproteases-2 [31]. 

To summarize, as stated by Sunagar *et al.* [32], in contrast to snake venoms rich in enzymatic toxins, scorpion venoms are dominated by peptide toxins of long or shorter chains, all having the CSab motif compatible with a great number of effects: toxins active on sodium channels (two types, alpha and beta), potassium channels—a family with a great diversity—and also chloride channels.

Most of the scorpion toxins have the common motif “CSab”, which raises the important question of a common ancestor for venom toxins and defensins, as defensins and toxins share a similar molecular weight and a consensus sequence [7]. 

The Buthidæ venoms are poor in enzymes. Only a hyluronidase and recently a metalloprotease have been identified in their venoms. The venoms of non-Buthidæ contain many other enzymes, but generally their stings are not dangerous for humans, except for the sting of *Hemiscorpius lepturus* (Hemiscorpiidæ) from Iran. The experimental toxicity of Buthidæ venoms varies according to the species. There is a good correlation between experimental tests and epidemiological observations. 

The toxicity of the scorpion venoms (LD 50 to mice) varies from 0.25 to 0.70 mg/kg [33]. Of course, the purified long toxins are more active: however, their toxicity is by far lower than the toxicity of bacterial toxins, in particular the clostridial toxins (Table 2). 

**Table 2:** Compared toxicities of some toxins and toxic agents, according to Mebs [34].

Toxins	LD50 (g/kg)	Mr
Botulinic toxin	0.00026	150,000
Batrachotoxin (amphibians)	2	538 *
Tetrodotoxin (fish)	9	319 *
Scorpions (Buthidæ)	10	7,000
Taipoxin (Elapid snake)	2	46,000
Notexin (Elapid snake)	25	13,500
-neurotoxins (Elapid snake)	75	7,800
d-tubocurarine (Plant)	200	696 *

Numbers with asterisks are alkaloids. Other toxins are venomous proteins.

The toxicity of the other toxins is more difficult to evaluate. The short toxins acting on potassium channels bind many cellular types, not only the excitable cells but also other cellular types such as leucocytes or hepatocytes. However, they do not seem toxic for mammals, except if injected by intrecerebral route. They are currently considered as potentiating agents of long toxins. Long toxins, which are responsible for the symptomatology of the scorpion envenomings, do not cross the blood brain barrier: the scorpion envenomings primarily affect peripheral nerve fibers and not nerve centers [35]. Short toxins do not seem to participate in the development of the symptoms of the envenomings, at least in mammals. However, sodium, potassium, and chloride channels are modified in their functioning or even blocked by their specific toxins. And as the potassium channels are largely distributed in the organism, the scorpion venom toxins blocking the potassium channels have been extensively studied.

After snakes, the scorpions represent the most dangerous venomous animals for humans, especially for children and young adolescents. The serotherapy for the treatment of scorpion stings has been discussed [13]. Epidemiological surveys suggest that man is particularly sensitive to scorpion Buthidæ venoms if the annual number of stings and the lethality are taken in account [36]. The most toxic venoms are those of the Buthidæ *Androctonus* sp. and *Leiurus quinquestriatus* (paleotropical species), *Tityus* sp. and *Centruroides* sp. (Neotropical species). It may be recalled that the lethal doses of their venoms in mice are about 0.25–0.30 mg/kg [33]. Long toxins, which are more powerful and present in an amount of about 4% of the dry weight of the venom are among the most dangerous for mammals (Table 2).

### 2.4. Discussion

The following questions come to mind: do other venoms contain toxins with a similar structure and do they have the same effects? The answer is affirmative: toxins with similar structures and functions are also found in the Cnidæ venoms. They are also active on membrane ion channels, especially sodium channels. The motif CSab is also found in the venom toxins of Cnidæ. It should also be noted that even though scorpions are among the most ancient terrestrial arthropods, the phylum Cnidæ appeared a long time before that, in the Cambrian. Cnidæ are at the forefront of primitive species of the living world. This model of toxins is also found in spider venoms, but their toxins often possess a slightly different ICK motif (Inhibitor Cystein Knot) near the CSab motif. Some snake venoms (*Crotalus durissus terrificus*, Crotalinæ) contain a myotoxin akin to the defensins, the crotamine (Crt) [37], which Fry believes belongs to the group of the most ancient snake venom proteins [38]. And, like the defensins, Crt shares the CSab motif and interacts with membrane lipids: the structure and the properties of Crt and vertebrate defensins hBD-2 type are homologous [39]. Venomous snakes are now considered to be primitive snakes: more generally, the venomous function in snakes is regressive. Besides, the family of CRISPs peptides (Cystein Rich Secreted Proteins) has diversified into many venom toxins. According to Fry (2005): “the toxin types where the ancestral protein was extensively cystein cross-linked were the ones that flourished into functionally diverse, novel toxin multigene families” [38]. As Crt belongs to the CRISPs, the structural relationship between venom toxins and defensins also concerns venomous snakes [39].

A next question is then raised: are there any other venoms, the toxins of which are structurally related to defensins? The answer is again affirmative: the venom of platypus. Only the platypus males are venomous and only produce venom during the mating phase. The majority of toxins in this venom has a similar structure to that of defensins [40], more precisely the structure of vertebrate defensins. Therefore, the structural relationship between venom toxins and defensins does not only concern scorpions, but also venomous vertebrates, snakes, and platypus.

These results support the hypothesis of Dufton [41] who believes that primitive mammals were originally venomous which gave them a small initial selective advantage. Indeed, the current living venomous mammals (monotremes, insectivores) are considered primitive forms of mammals. Given all these results, it is conceivable that the venomous function and the innate immunity are closely related, and, therefore, the scorpion venom toxins and the defensins would have a common ancestor. This is precisely the result of the paper of Cao *et al.* [42] who write: “Remarkably, our results not only point to the monophyly of the neurotoxin and defensin genes in *Mesobuthus martensii*, but it is most likely that Na^+^-Toxins diverged first from the common ancestor of K^+^-Toxins, Cl-Toxins and defensin genes that subsequently diversified and formed separate families”. Considering this paper, Andreotti and Sabatier [43] have noted that the results of Cao *et al.* [42] explain the fact that scorpions are not sensitive to their own venom as demonstrated by Legros *et al.* [44].

This generalization is plausible even if some molecular data are still lacking for many toxins: Zhu *et al.* (2014) have recently obtained the experimental conversion of a defensin in a neurotoxin [45]. In fact the existence of an antibacterial scorpion peptide with a similar structure to a curarizing cone toxin that presents the same curarizing activity already was known [16]. Conceptually, in the hypothesis of a venomous function ensuring animal protection, a differentiation of the basic protective self-function into a venomous function raises no intellectual objection. The hypothesis of the involvement of the venomous function in nutrition, especially in carnivorous predator species, would become an additional differentiation, as venoms could be important in the survivability of primitive venomous mammal species, as suggested by Dufton [41].

Scorpions, which are panchronic animals, have a venom with a very simple composition, at least in the Buthidæ family, considering the structure and the effects of the different toxins (short peptides with the same structural motif CSab, all acting on ionic channels). The scorpion venoms could be considered as an archetype of primitive venoms, as the toxins are similar to defensins or sometimes to antimicrobial peptides, their genes constituting a monophyletic group in the scorpion *Mesobuthus martensii* (Buthidæ) according to Cao *et al.* [42]. But the importance of the venom toxins for developing new drugs must not be underestimated, due to their pharmacological advantages including their high activity, high specificity, lack of accumulation in organisms, low immunogenicity, and limited targets. Claude Bernard (1813–1878), a glorious visionary, already wrote: “The real scientific basis of the therapeutics must be given by the knowledge of the physiological action of drugs or poisons which is exactly the same thing” [46].
